# *Pseudarthrobacter oxydans* RH60-mediated rhizobacterial interactions bidirectionally regulate cucumber root-knot disease

**DOI:** 10.3389/fmicb.2026.1926380

**Published:** 2026-09-11

**Authors:** Peng Yu, Yanru Yu, Huihui Li, Yingmei Li, Xiaoli Guo, Mengxin Zhao, Peiyao Wei, Gaofeng Wang

**Affiliations:** 1 National Key Laboratory of Agriculture Microbiology, Huazhong Agricultural University, Wuhan, China; 2 Key Laboratory of Plant Pathology of Hubei Province, College of Plant Science & Technology, Huazhong Agricultural University, Wuhan, China; 3 Shaanxi Key Laboratory of Plant Nematology, Bio-Agriculture Institute of Shaanxi, Xi’an, China

**Keywords:** continuous cropping obstacle, microbe-derived VOCs, rhizobacterial interaction, root exudates, root-knot nematode

## Abstract

Intensive cucumber continuous cropping disrupts rhizosphere balance and triggers severe *Meloidogyne incognita* infestation. Cucumber-pepper rotation alleviates this via recruiting the rhizobacteria *Pseudarthrobacter oxydans* RH60. However, the mediating role of rhizobacterial interaction networks remains unclear. Here, we clarified the bidirectional regulatory mechanism of this nematode disease via RH60-centered rhizobacterial interactions. Rhizobacteria from the rhizospheres of continuously cropped cucumber and rotated pepper were firstly isolated. Their effects on RH60 growth were screened via fermentation filtrate and filter disc assays. Target strains were identified by 16S rRNA gene sequencing and phenotypic characterization. *Myroides odoratus* P69 (from pepper rhizosphere) was identified as an RH60-synergistic strain and *Bacillus toyonensis* RH119 (from cucumber rhizosphere) as an RH60-antagonistic strain. Data from growth promotion and biofilm formation assays showed that pepper root exudates specifically recruited P69, while cucumber root exudates specifically enriched RH119. Two-compartment petri dish co-culture and gas chromatography–mass spectrometry (GC–MS) assays were performed. The results indicated that RH119 inhibited RH60 growth by releasing volatile organic compounds (VOCs) (predominantly isoamyl isovalerate and isopropyl 2-methylbutyrate). Quantitative real-time PCR (qPCR) was used to quantify RH60 rhizosphere colonization, and pot experiments were conducted to verify disease control efficacy. P69 enhanced RH60 colonization capacity and disease-suppressive effect, thereby promoting cucumber root growth. In contrast, RH119 reduced RH60 colonization and exacerbated the nematode disease. These results demonstrate the root exudates and microbe-derived VOCs jointly mediate RH60-centered interactions. These interactions drive bidirectional nematode disease regulation under continuous cropping and rotation, providing insights for sustainable cucumber production.

## 1 Introduction

Intensive facility agriculture has become a core model for global vegetable production. However, its sustainability is increasingly threatened by continuous cropping-induced ecological degradation of agricultural ecosystems (Shen et al., 2021; Walker et al., 2023). In cucumber cultivation, continuous cropping disrupts the balance of the rhizosphere ecosystem. This disruption leads to the accumulation of autotoxic substances, the breakdown of soil microbial community stability, and enhanced pathogenicity of root-knot nematodes (Zhou et al., 2021; Li et al., 2024; Tian et al., 2025). As a destructive soil-borne pathogen with broad host range (Khan et al., 2023), the root-knot nematode *Meloidogyne incognita* causes severe yield losses in cucumber, and its outbreaks indicate a rhizosphere ecosystem function decline (Zhang et al., 2024; Chen et al., 2025).

Crop rotation, a classic ecological management practice, alleviates continuous cropping obstacles by modifying soil physicochemical properties and reshaping microbial community composition. These changes restore ecosystem services such as disease suppression and nutrient cycling (Bybee-Finley et al., 2024; Gun et al., 2025). A previous study by Tian et al. (2025) confirmed that cucumber-pepper rotation recruits the rhizobacterium *Pseudarthrobacter oxydans* RH60. This strain contributes to rhizosphere ecosystem regulation by degrading phenolic acids (allelochemicals in root exudates) (Tian et al., 2025). RH60 also suppress the root-knot nematode *M. incognita* (Tian et al., 2025). However, in ecological systems, no single species acts in isolation. The function of key microorganisms, such as the strain RH60, depends on their interactions with other community members. However, how these microbial interaction networks mediate the response of the rhizosphere ecosystem to continuous cropping/rotation remains unelucidated. This gap limits our ability to translate microbial findings into ecologically sound management strategies for agricultural systems.

Rhizobacteria are critical drivers of rhizosphere ecosystem function, shaping plant disease resistance and soil health through competitive, synergistic, and metabolite-mediated interactions (Olenska et al., 2020; Munoz-Ucros et al., 2021). Root exudates, as “ecological signals” from plants to the rhizosphere, selectively recruit beneficial or pathogenic microorganisms. This process is not random but a plant’s adaptive strategy to regulate rhizosphere ecosystem structure (Hu et al., 2021; Chai and Schachtman, 2022; Santoyo, 2022). For example, potatoonion root exudates induce tomato plants to modify their root exudate chemistry, thereby recruiting beneficial *Bacillus* sp. to form a disease-suppressive rhizosphere microbiome that protects against *Verticillium dahlia* (Zhou X. A. et al., 2023). In contrast, potato continuous cropping results in leads to the accumulation of harmful fungi by changing the metabolic pathway of root exudates (Xing et al., 2024). Similarly, microbial volatile organic compounds (VOCs), as low-molecular-weight signaling molecules, are key mediators of interspecific communication in soil, influencing pathogen survival and plant immunity as part of the rhizosphere’s chemical ecology (Singh et al., 2021; Ravelo-Ortega et al., 2023). *Bacillus* sp. NYG5, for instance, secretes VOCs to promote biomass of *Arabidopsis thaliana*, *Nicotiana tabacum*, and *Cucumis sativus* by activating plant metabolic pathways, restructuring rhizosphere microbiota, decreasing soil humic substances, and via direct growth promotion (Sudakov et al., 2025). *B. subtilis* 0618A-derived VOCs, with 2,4-di-tert-butylphenol (2,4-DTBP) as the primary active component, potently suppress mycelial growth and sclerotia germination of *Sclerotium rolfsii*, exhibiting high efficacy and safety in the biocontrol of peanut southern blight (Cheng et al., 2025). However, how VOCs mediate interactions between key rhizobacteria and other community members. Furthermore, the mechanism by which VOCs thereby regulate the overall disease-suppressive function of the rhizosphere ecosystem, remains unclear.

Crop rotation alters root exudate profiles, thereby restructuring rhizobacterial interaction networks (Stirnemann and Sasse, 2025). Building on this ecological framework and the prior observation that rhizobacterial strain RH60 mitigates continuous cropping obstacles in cucumber under a cucumber-pepper rotation system (Tian et al., 2025), three hypotheses were tested herein: (1) Continuous cucumber cropping disrupts the rhizosphere ecosystem by enriching RH60-antagonistic bacteria, which suppress RH60 functionality and exacerbate root-knot nematode disease; (2) Pepper rotation restores rhizosphere ecosystem balance by enriching RH60-synergistic bacteria, thereby enhancing RH60’s disease-suppressive capacity; (3) Root exudates (plant-derived ecological signals) and VOCs (rhizobacteria-derived chemical signals) jointly mediate these RH60-centered rhizobacterial interactions; They serve as “linkages” between plant community shifts (pepper-cucumber rotation) and rhizosphere ecosystem function (root-knot nematode disease suppression on cucumber).

This study goes beyond investigations into the mechanism of individual microbial strains. It advances our understanding of the ecological processes underlying continuous-cropping obstacles and rotation-mediated rhizosphere remediation. Specifically, we elucidate the role of RH60-centered rhizobacterial interaction networks in the bidirectional regulation of cucumber root-knot disease under continuous cropping and rotation. The goal is to provide theoretical support for sustaining agricultural ecosystem stability and alleviating cucumber continuous cropping obstacles.

## 2 Materials and methods

### 2.1 Plant and nematode culture

Cucumber (*Cucumis sativus* cv. Bendiwang) and pepper (*Capsicum annuum* cv. 8,819 Xianjiao) seeds were germinated at 28 °C in the dark, and transplanted to sterilized nutrient soil (10 cm × 10 cm × 8 cm pots) under greenhouse conditions (25 °C, 16 h light/8 h dark). The root-knot nematode, *M. incognita* was maintained on cucumber. Eggs were extracted via 0.8% NaClO, purified by 40% sucrose centrifugation, and incubated at 28 °C for 3–7 days to obtain second-stage juveniles (J2s) (Tiwari et al., 2017). All the following experiments involving cucumber and pepper plants were conducted under the aforementioned controlled environmental conditions.

### 2.2 Isolation and purification of rhizosphere bacteria

Rhizosphere soil samples of pepper (from pepper continue cropping fields) and cucumber (from cucumber continue cropping fields) were collected as described by Tian with minor modifications (Tian et al., 2025). Briefly, pepper and cucumber roots were shaken in sterile PBS for 20 min, and the shaking was repeated once. Subsequently, the root samples were sonicated in sterile water for 10 min to dislodge rhizosphere soil, and the resulting suspension was serially diluted to 10^−3^, 10^−4^, 10^−5^, and 10^−6^. Aliquots of 100 μL were spread on different media and incubated at 28 °C for 2 days. These media included LB (Luria-Bertani), TSB (Tryptic Soy Broth), TYG (Tryptone-Yeast extract-Glucose), YEM (Yeast Extract Mannitol), M715 (Mannitol-Glutamate Medium 715), Medium 408 and PDA (Potato Dextrose Agar). Colonies with distinct morphologies were streaked onto LB medium for purification, and the purified strains were stored in 50% glycerol at −80 °C for subsequent use.

### 2.3 Screening of RH60 growth promoting and inhibiting strains

To screen pepper-derived rhizobacterial strains capable of promoting RH60 growth, the isolated rhizobacterial strains were pre-cultured in 1 mL LB medium at 28 °C with shaking at 180 r/min to an OD_600_ of 0.8. Subsequently, the bacterial suspensions were inoculated into fresh LB medium at a volume ratio of 1:100 (strain suspension/LB medium). The cultures were incubated with shaking (180 r/min) at 28 °C for 48 h. The cultures were then centrifuged at 10,000 × g for 10 min, and the supernatants were filtered through a 0.22 μm membrane to obtain fermentation filtrates. Subsequently, the fermentation filtrates were added to 1/5 LB medium at a volume ratio of 1:10 (fermentation filtrates/LB), followed by inoculation with 10 μL of RH60 suspension (OD₆₀₀ = 0.8) and cultivation at 28 °C. After 16 h of incubation, the OD₆₀₀ value was measured. Meanwhile, 100 μL of 10^3^-fold diluted RH60 suspension was streaked onto LB agar plates, and the colony-forming units (CFUs) were recorded after 16 h of cultivation at 28 °C. LB medium without fermentation filtrate served as the negative control, and all experiments were repeated six times.

For screening rhizobacterial strains exhibiting RH60-inhibiting activity from cucumber, the strains isolated in this study and that isolated previously and kept in our laboratory were assayed. 100 μL RH60 (OD₆₀₀ = 0.8) was spread on LB plates, and sterile filter discs (6 mm diameter) with 10 μL rhizobacterial strains (OD₆₀₀ = 0.8) were placed on plates at the same time. After 24 h of cultivation at 28 °C, inhibition zone diameters were measured. All experiments were repeated three times.

### 2.4 Effects of pepper and cucumber root exudates on the growth and biofilm formation of candidate strains

To determine the recruitment effect of root exudates from both pepper and cucumber on candidate strains, the effects of these root exudates on bacterial growth and biofilm formation were evaluated. Firstly, seedlings at the one-true-leaf stage were hydroponically cultured in sterile Hoagland’s solution for 24 h. Root exudates were sterile-filtered, freeze-dried, and stored at −80 °C as described (Williams et al., 2021). A 10 μL aliquot of candidate bacterial suspension (OD₆₀₀ = 0.6) was inoculated into 10 mL TSB supplemented with 100 μg/mL root exudates (control group: TSB without root exudates). After a shaking incubation (180 r/min) at 28 °C for 12 h, bacterial growth was determined by measuring OD₆₀₀ values. Each experiment was performed in triplicate.

Then, biofilm formation of candidate strains was assessed via crystal violet staining as described by Tian et al. (2025) with minor modifications. Using water substituting root exudates as the control group. Briefly, 10 μL of candidate bacterial suspension (OD₆₀₀ = 0.6) was inoculated into TSB containing 100 μg/mL root exudates. Following homogenization, the mixture was dispensed into a 96-well plate (100 μL per well) and statically incubated at 28 °C for 24 h. The culture supernatant was then aspirated, and the wells were rinsed three times with 200 μL of PBS buffer. For fixation, 100 μL of methanol was added to each well and incubated for 15 min, after which the methanol was aspirated and the plate was air-dried at room temperature. Subsequently, 100 μL of 1% crystal violet solution was added for staining (5 min), followed by three rinses with ddH₂O and air-drying at room temperature. Finally, 100 μL of 33% glacial acetic acid was added to each well, and the plate was statically incubated at 30 °C for 30 min to dissolve the crystal violet. The absorbance at 562 nm was measured using a microplate reader. Five technical replicates were established per bacterial strain for biofilm formation assays.

### 2.5 Compatibility analysis among candidate strains

The compatibility between different candidate strains was evaluated using the filter disc diffusion assay. Briefly, 100 μL of a bacterial suspension (OD₆₀₀ = 0.6) of one candidate strain was spread evenly on LA (Luria-Bertani agar) plates. Sterile filter discs (diameter: 6 mm) impregnated with 10 μL of bacterial suspension (OD₆₀₀ = 0.6) of other candidate strains were then placed on the inoculated agar surface. Plates were incubated at 28 °C for 24 h, after which the diameter of inhibition zones (if present) around each filter disc was measured using a vernier caliper. The absence of an inhibition zone indicated mutual compatibility between the tested strain pairs, while the presence of a clear zone suggested antagonistic interactions. Each strain combination was tested in three biological replicates.

### 2.6 Strain identification

For genus-level identification of the candidate strains, molecular identification based on 16S rRNA gene sequencing was conducted. The 16S rRNA gene was amplified via PCR using the universal bacterial primers, 27F (5′-AGAGTTTGATCCTGGCTCAG-3′) and 1492R (5′-ACGGCTACCTTGTTACGACTT-3′) (Li et al., 2022). The obtained PCR amplicons were sequenced, and the resulting sequences were subjected to BLASTn analysis against the NCBI GenBank database for homologous sequence alignment. Phylogenetic trees were constructed using MEGA X software with the neighbor-joining (NJ) method, and bootstrap validation was performed with 1,000 replicates to assess tree reliability.

For further species-level identification, phenotypic characterization including colony morphology observation and Gram staining was conducted. For colony morphology analysis, bacterial suspensions were spread evenly on LA media and incubated at 28 °C for 24 h. Colony characteristics (e.g., shape, color, size, margin, and texture) were recorded via visual observation. Gram staining was performed using a Gram stain kit according to its protocol (Solarbio, China), and stained cells were observed under a light microscope to determine the Gram reaction (positive/negative) and cell morphology.

### 2.7 Impact of target strains on RH60 colonization in cucumber rhizosphere

We evaluated the effect of target strains on RH60 colonization in the cucumber rhizosphere. The absolute abundance of RH60 in rhizosphere soil was quantified via quantitative real-time PCR (qPCR) as described previously (Tian et al., 2025). Briefly, a RH60-specific gene fragment was amplified from RH60 genomic DNA using the specific qPCR primers q60F (5′-CCTCAAAGGCGAAAAAGGGC-3′) and q60R (5′-GGCCGAGATTTCAGGACCAA-3′), and cloned into the pTOPO-Blunt vector. A standard curve was then generated using the recombinant vector for absolute quantification. Cucumber seedlings at the one-true-leaf stage were cultured in sterile soil-filled pots at the controlled condition as described above. Two experimental groups were established: cucumber seedlings inoculated with *M. incognita* (1,200 eggs/J2s per pot) and non-inoculated seedlings, both of which were co-inoculated with target strains and RH60 simultaneously. Two control groups were included: a non-bacterial control (without target strains or RH60 inoculation) and a RH60-only control (inoculated with RH60 alone). Rhizosphere soil samples were collected at 7 and 30 days post-inoculation (dpi), and total soil DNA was extracted. The total absolute abundance of RH60 (including viable and dead RH60) was quantified by qPCR using the specific primers q60F and q60R. Each sample was analyzed with three technical replicates.

### 2.8 Effect of target strains on RH60-mediated suppression of cucumber root-knot nematode disease

Cucumber seedlings at the one-true-leaf stage were subjected to different bacterial inoculation treatments via the root-drenching method: (1) 15 mL of target strain suspension (OD₆₀₀ = 1) alone; (2) 15 mL of RH60 suspension (OD₆₀₀ = 1) alone; (3) 15 mL of mixed suspension of target strain and RH60 (each at OD₆₀₀ = 1, v:v = 1:1). Two days post-bacterial inoculation, 1,200 eggs and J2s of *M. incognita* per pot were inoculated. Seedlings drenched with sterile water instead of bacterial suspension served as the blank control. At 30 dpi of *M. incognita*, the following parameters were determined: (1) Plant growth indicators: root length, root fresh weight, shoot length, and shoot fresh weight; (2) Root-knot index (RKI): graded on a 0–4 scale as described (Krusberg and Nielsen, 1958): 0 = no galls; 1 = 1–25% of root system galled; 2 = 26–50% galled; 3 = 51–75% galled; 4 = 76–100% galled; (3) Rhizosphere soil nematode density: total number of *M. incognita* (eggs and J2s) per 100 mL of rhizosphere soil. Each treatment included 12 biological replicates for the determination of growth indicators and root-knot index, and 3 biological replicates for rhizosphere nematode density quantification.

### 2.9 Screening and identifying target strain-derived metabolites affecting RH60 growth

#### 2.9.1 Thermostability and liposolubility assays of bioactive metabolites

To characterize the physicochemical properties of bioactive metabolites from target strains that modulate RH60 growth, thermostability and liposolubility assays were performed. Briefly, sterile fermentation filtrates of target strains were prepared as described in Section 2.4. The filtrates were subjected to two distinct treatments: (1) Thermostability test: Filtrates were incubated at 80 °C for 1 h to evaluate the heat resistance of bioactive components; (2) Lipsolubility test: Filtrates were extracted with methanol (v/v = 1:1) to assess the lipid solubility of bioactive components.

The effects of treated fermentation filtrates on RH60 growth were determined using the method described in Section 2.4. Sterile LB broth without fermentation filtrate served as the negative control, while untreated sterile fermentation filtrate was used as the positive control.

#### 2.9.2 Two-compartment petri dish co-cultivation assay for volatile organic compounds (VOCs)-mediated RH60 growth regulation

A two-compartment Petri dish co-cultivation assay was conducted to investigate the role of target strain-derived VOCs in regulating RH60 growth (allowing volatile diffusion without direct bacterial contact). Briefly, 100 μL of target strain suspension (OD₆₀₀ = 1) and 100 μL of RH60 suspension (OD₆₀₀ = 0.8) were inoculated onto the two separate compartments of LA plates, respectively. LA inoculated with sterile water (instead of target strain) served as the negative control. All plates were statically incubated at 28 °C for 48 h, and RH60 growth on LA was recorded. Each treatment included six biological replicates.

#### 2.9.3 Identification of bioactive VOCs inhibiting RH60 growth via gas chromatography-mass spectrometry (GC-MS)

We aimed to identify the bioactive VOCs from target strains that inhibit RH60 growth. Target strains (initial OD₆₀₀ = 1) were inoculated into sterile LB broth at an inoculation ratio of 1:20 (v/v, bacterial suspension: LB medium). The cultures were then transferred to 20 mL sterile sealed headspace vials. The vials were incubated at 28 °C for 48 h to induce VOCs production. Volatile compounds were collected using solid-phase microextraction (SPME) and analyzed by GC–MS. The chromatographic and mass spectrometric conditions were optimized as follows:

Chromatographic conditions: A HP-5 quartz capillary column (30.0 m × 0.25 mm × 0.25 μm) was used. High-purity helium (>99.999%) served as the carrier gas at a constant flow rate of 1 mL/min. The injection port temperature was set at 230 °C, and split injection mode was employed with a split ratio of 10:1. The column temperature program was: initial hold at 50 °C for 2 min, ramp to 180 °C at 6 °C/min (held for 1 min), and final ramp to 250 °C at 10 °C/min (held for 3 min) to ensure complete elution of VOCs.

Mass spectrometric conditions: An electron impact (EI) ion source was used, with the ion source temperature at 230 °C, GC–MS interface temperature at 230 °C, and quadrupole temperature at 150 °C. The electron energy was 70 eV, and the mass scan range was 30–450 m/z.

Qualitative analysis of detected compounds was performed by matching mass spectra with the NIST 17 mass spectral library. Retention time and characteristic mass fragmentation patterns were also verified for compound identification. Quantitative analysis was conducted using the peak area normalization method, where the relative content of each compound was expressed as the percentage of its peak area relative to the total peak area of all detected compounds.

#### 2.9.4 Verification of the inhibitory role of bioactive VOCs via split-plate assays

To further validate the inhibitory effect of bioactive VOCs (identified via GC–MS in Section 2.9.3) produced by target strains on RH60 growth, split-plate assays were performed using corresponding chemical reference standards (purchased from Macklin Chemical Co., Ltd. or Aladdin Chemical Co., Ltd.). Briefly, LA plates (split-plate design) were used for the assay. Different volumes (10, 50, 100 and 250 μL) of reference standard solutions (0.86 mg/mL) were added to one compartment of each split-plate, while 100 μL of RH60 suspension (OD₆₀₀ = 0.8) was uniformly spread on the other compartment (ensuring no direct contact between the standard solution and RH60). Sterile water was used as the negative control (substituted for reference standards). All plates were statically incubated at 28 °C for 48 h to allow VOCs diffusion, and RH60 growth was assessed by the number of CFUs.

The contribution of each bioactive VOC to the target strain-mediated inhibition of RH60 growth was evaluated based on two key criteria: (1) the relative content of the VOC in the target strain’s VOC profile; and (2) the inhibitory activity against RH60 growth (stronger inhibitory effect indicating higher bioactivity). A VOC with lower relative content and stronger inhibitory activity was considered to have a greater contribution to the overall inhibitory effect of the target strain on RH60. Three biological replicates were set up.

### 2.10 Data analysis

All data were expressed as mean ± standard error (SE). Student’s *t*-test was used for pairwise comparisons. One-way analysis of variance (ANOVA)/two-way ANOVA followed by Tukey’s honestly significant difference (HSD) test was applied for other multiple group comparisons. Values with *p* < 0.05, *p* < 0.01, or *p* < 0.001 were considered statistically significant. Statistical analyses were performed using SPSS 20.0 software, while tables and graphs were generated with Office 2021 and GraphPad Prism 10.0 software.

## 3 Results

### 3.1 10 candidate target rhizobacteria affecting RH60 growth were isolated from pepper and cucumber

We aimed to elucidate rhizobacterial interactions centered on *P. oxydans* RH60. These interactions mediate bidirectional regulation of cucumber root-knot nematode disease under cucumber continuous cropping and pepper-cucumber rotation systems. Hence, strains were isolated and screened (Supplementary Figure S1). First, 116 rhizobacterial strains (P1-P116) from pepper rhizosphere and 136 strains (C1-C136) from cucumber rhizosphere were isolated (Supplementary Table S1). To expand the screening pool, 124 previously isolated and preserved cucumber rhizobacterial strains (RH1-RH124) were included. This resulted in a total of 260 cucumber-derived and 116 pepper-derived strains (Supplementary Table S1). All strains were initially screened for their effects on RH60 growth. Data showed that 13 pepper-derived strains promoted RH60 growth, and 17 cucumber-derived strains inhibited it (Supplementary Table S1), which were designated as putative candidates.

**Figure 1:**
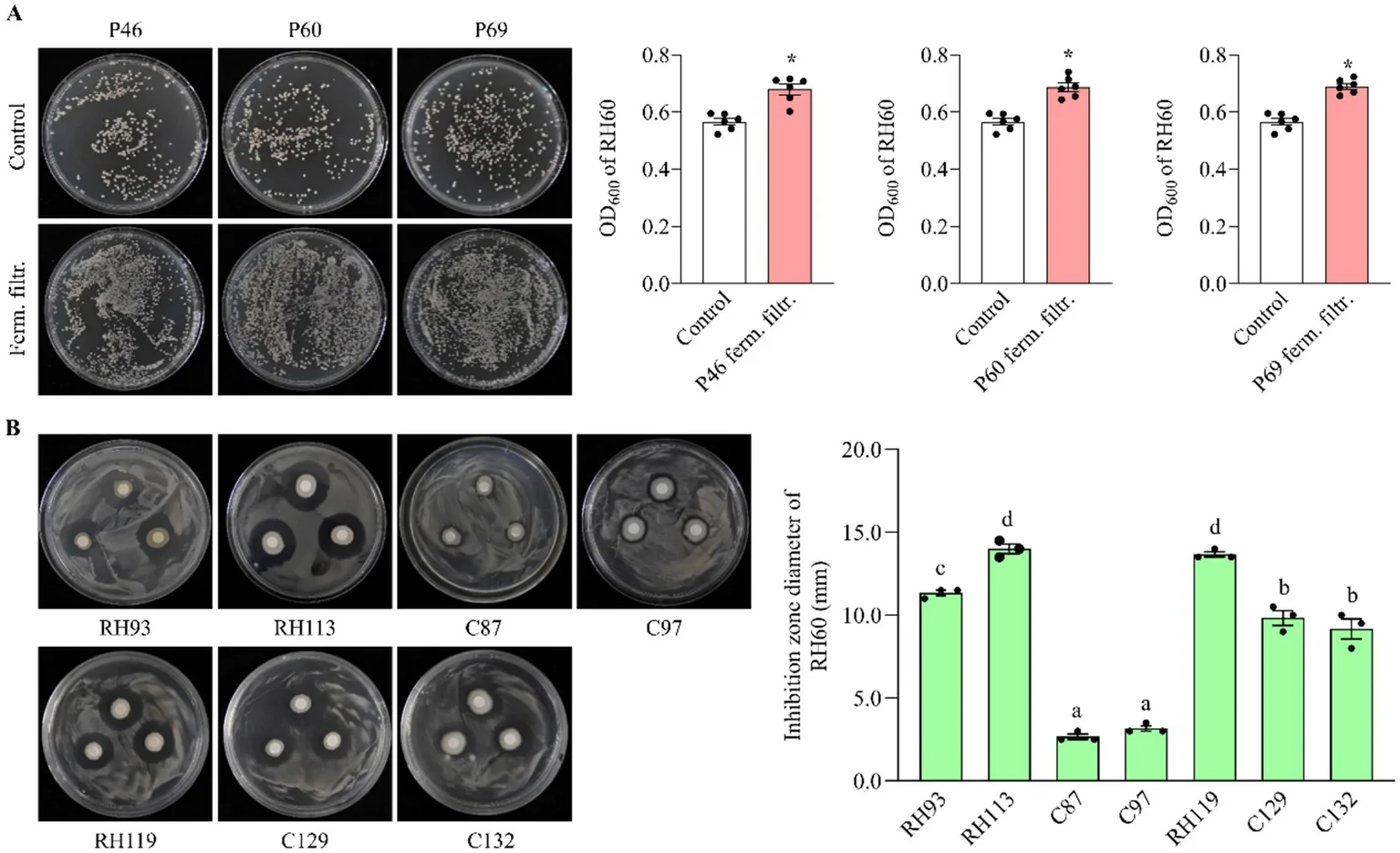
Evaluation of the effects of rhizosphere bacterial strains on the growth of target strain RH60. **(A)** Growth-promoting effect assay: RH60 was inoculated onto LB medium supplemented with the fermentation filtrate (Ferm. filtr.) of tested strains; the colony forming units (CFUs) and OD₆₀₀ values of RH60 were determined after 16 h of cultivation, with LB medium without fermentation filtrate set as the blank control. **(B)** Growth-inhibition effect assay: 100 μL of RH60 suspension (OD₆₀₀ = 0.8) was uniformly spread on LB solid medium plates; sterilized and dried sterile filter paper discs (containing 10 μL of rhizosphere bacterial suspension (OD₆₀₀ = 0.8) per paper discs) were placed on the plate surface; the plates were incubated at 28 °C for 24 h, and the formation of inhibition zones around the filter paper discs was observed and recorded. Values are mean ± SE, *n* = 6 **(A)** and 3 **(B)**. * (Student’s *t* test) and different letters (one-way ANOVA with post-hoc Tukey HSD test) indicate significant difference at *p* < 0.05 (**A,B**, respectively).

These 30 candidates were further identified at the genus level via 16S rRNA gene sequencing. The result showed that the 13 growth-promoting strains belonged to *Myroides* spp., *Citrobacter* spp., and *Arthrobacter* spp., while the 17 inhibitory strains were classified into *Bacillus* spp. and *Pseudomonas* sp. Based on genus affiliation, 3 growth-promoting (from pepper) and 7 inhibitory (from cucumber) strains were selected for subsequent screening (Supplementary Table S2). Among these, the growth-promoting strains P46, P60, and P69 exhibited comparable RH60-promoting activity (Figure 1A), and the inhibitory strains RH119 and RH113 showed the strongest RH60-inhibitory effects (Figure 1B).

### 3.2 P69 (pepper-derived) and RH119 (cucumber-derived) are identified as the target strains

**Figure 2:**
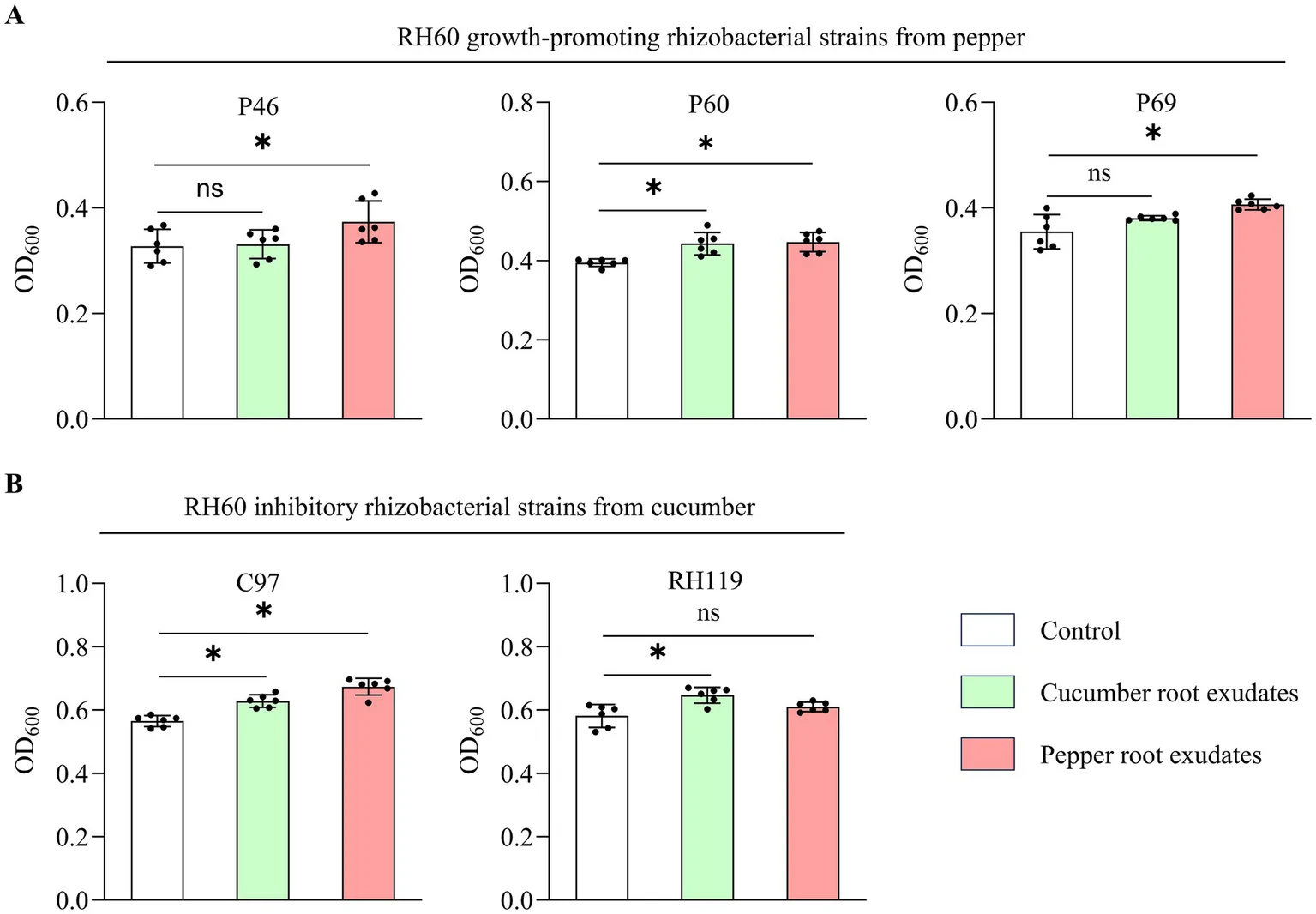
The growth responses of RH60 growth-promoting and inhibitory strains to root exudates of cucumber and pepper. To evaluate the growth responses of growth-promoting (P46, P60, P69) and RH60 inhibitory (C97, RH119) strains toward cucumber and pepper root exudates, the OD_600_ values of RH60 growth-promoting **(A)** and inhibitory **(B)** strains were determined after 12 h of cultivation at 28 °C in LB media supplemented with 100 μg/mL of the aforementioned root exudates, with LB media without root exudates as blank controls. Values are mean ± SE, *n* = 6. * and “ns” indicates significant and no significant difference (student’s *t* test) at *p* < 0.05.

**Figure 3:**
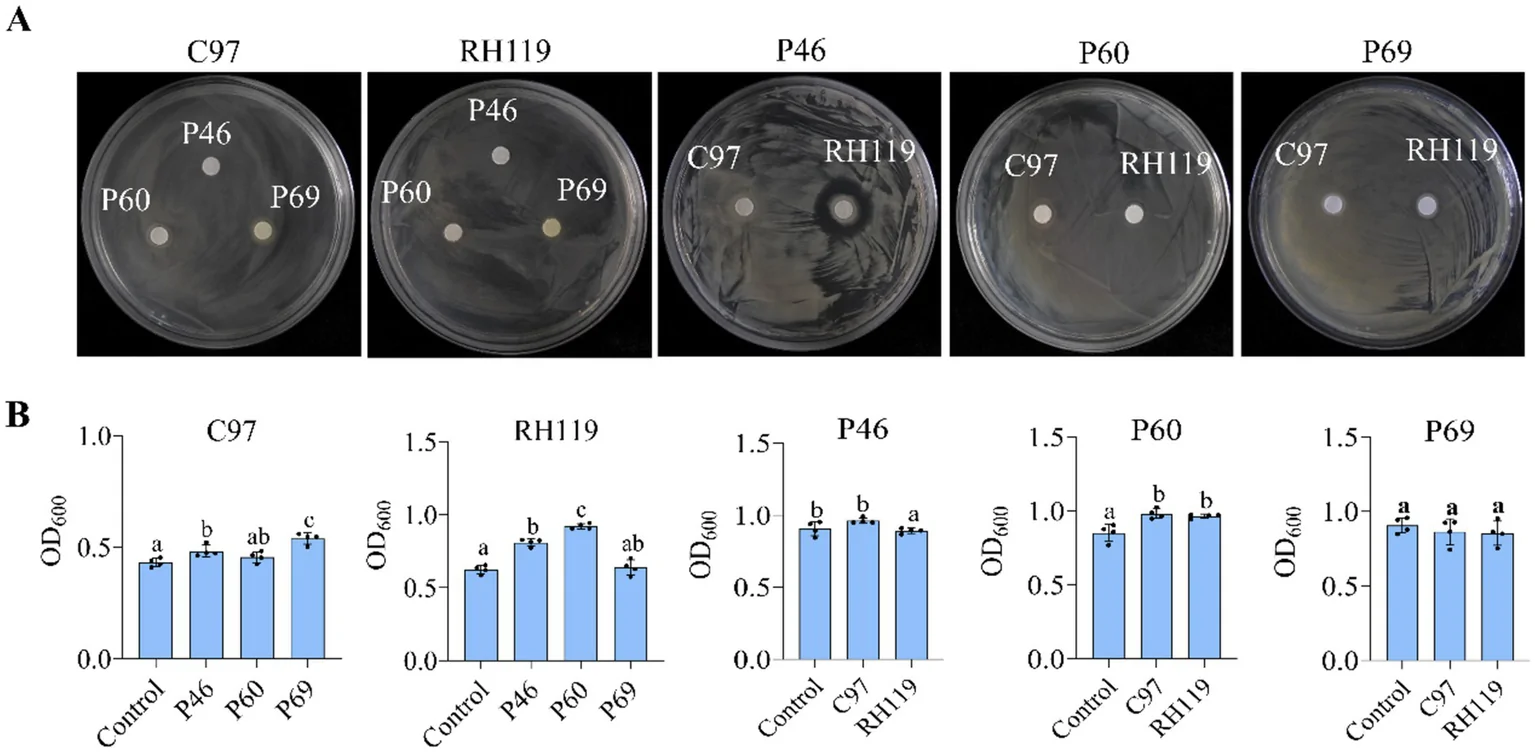
Interspecific compatibility analysis between RH60 inhibitory and growth-promoting strains. To assess interspecific compatibility between RH60 inhibitory and growth-promoting strains, two experiments were performed: **(A)** antagonistic effects among the strains (visually marked yellow or white) were evaluated on LA media **(B)** each strain was cultured in TSB media supplemented with 10% fermentation filtrates of the other strains, and OD₆₀₀ values were determined after 12 h of incubation at 28 °C, with TSB media without fermentation filtrates as blank controls. Values are mean ± SE, *n* = 3 **(B)**. Different lowercase letters indicate significant differences (one-way ANOVA with post-hoc Tukey HSD test) at *p* < 0.05 (**B**).

Next, the recruitment specificity of root exudates to these strains was evaluated based on chemotaxis, growth promotion, and biofilm formation assays. Pepper root exudates recruited all three growth-promoting strains, while cucumber root exudates recruited C97 and RH119 (Supplementary Figure S2). Further analysis revealed that cucumber, but not pepper, root exudates promoted RH119 growth, while pepper, rather than cucumber, root exudates stimulated the growth of P46 and P69 (Figure 2). To fulfill the criterion that target strains must not mutually promote each other’s growth, inter-strain interactions were tested. Data indicated that P46 and P60 both promoted RH119 growth (Figure 3), and were thus ruled out. This identified P69 (RH60 growth-promoting) and RH119 (RH60 inhibitory) as the final target strains. Finally, P69 and RH119 were identified as *M. odoratus* and *B. toyonensis*, respectively, based on Gram staining, colony morphology, and phylogenetic analysis of 16S rRNA gene sequences (Supplementary Figure S3).

### 3.3 P69 and RH119 exert opposite functions in regulating RH60-mediated reduction of cucumber root-knot nematode disease severity

**Figure 4:**
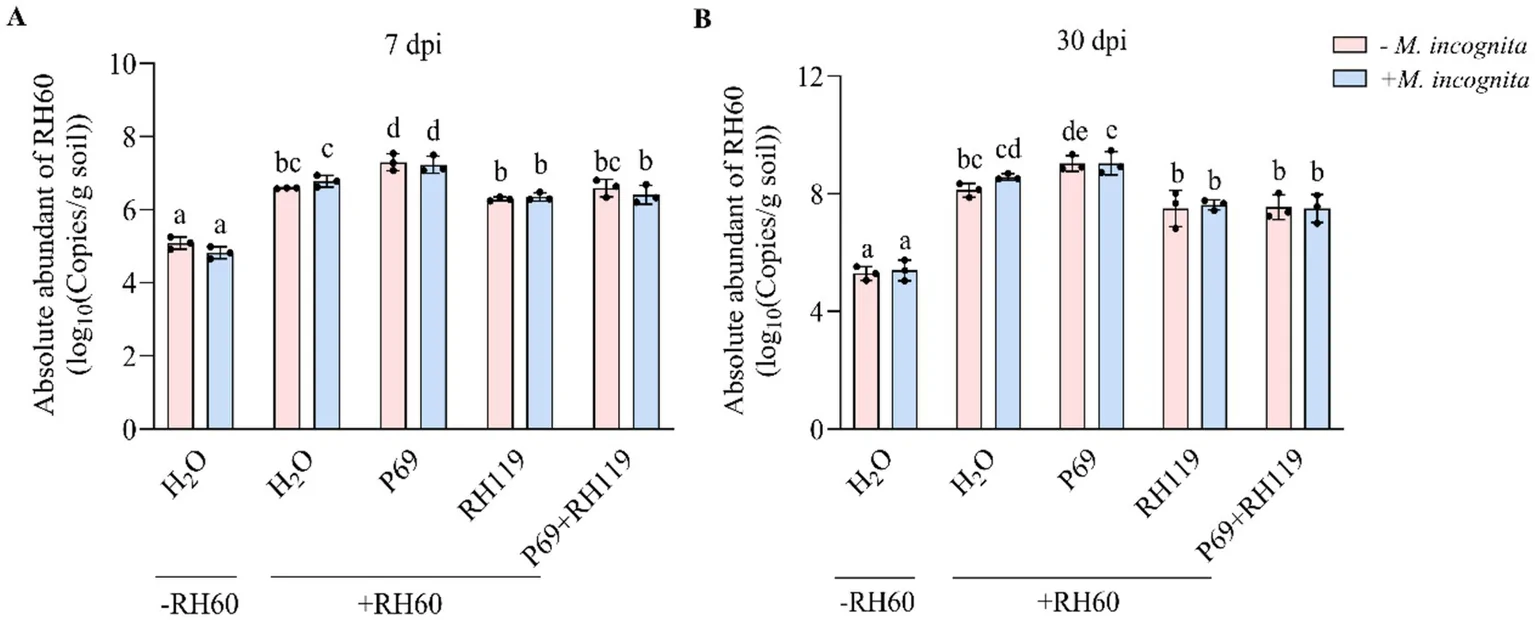
Effects of P69, RH119 and their combination on RH60 colonization in cucumber rhizosphere. To evaluate the effects of P69, RH119, and their combination on RH60 colonization in cucumber rhizosphere (with or without *M. incognita* inoculation), the absolute abundance of RH60 (including viable and dead RH60) was determined in rhizosphere soil at 7 days **(A)** and 30 days **(B)** after bacterial inoculation. Cucumber plants without bacterial inoculation served as controls, and bacterial suspensions were co-inoculated with *M. incognita*. Values are mean ± SE, *n* = 3. Different lowercase letters indicate significant differences (two-way ANOVA with post-hoc Tukey HSD test) at *p* < 0.05.

We clarified the roles of P69 and RH119 in RH60-mediated suppression of *M. incognita*-caused nematode disease on cucumber. First, their impacts on RH60 colonization in the cucumber rhizosphere were evaluated. A qPCR standard curve was established using RH60 copy number versus Ct values (Supplementary Figure S4). At 7 dpi of bacteria, P69 increased the absolute abundance of RH60 in rhizosphere soil compared to sole RH60 inoculation. This promoting effect was observed regardless of *M. incognita* infection (Figure 4A). This promoting effect, however, was only observed in nematode-non-inoculated plants at 30 dpi (Figure 4B). In contrast, RH119 reduced RH60 abundance in the rhizosphere of nematode-infected cucumbers at both time points, while no such reduction was detected in non-infected plants (Figure 4). These results indicate that P69 and RH119 play opposite roles in regulating RH60 rhizosphere colonization. Notably, co-inoculation of P69 and RH119 had no significant effect on RH60 abundance relative to sole RH60 inoculation (Figure 4), suggesting that RH119 antagonizes the promotion effect of P69 on RH60 colonization.

**Figure 5:**
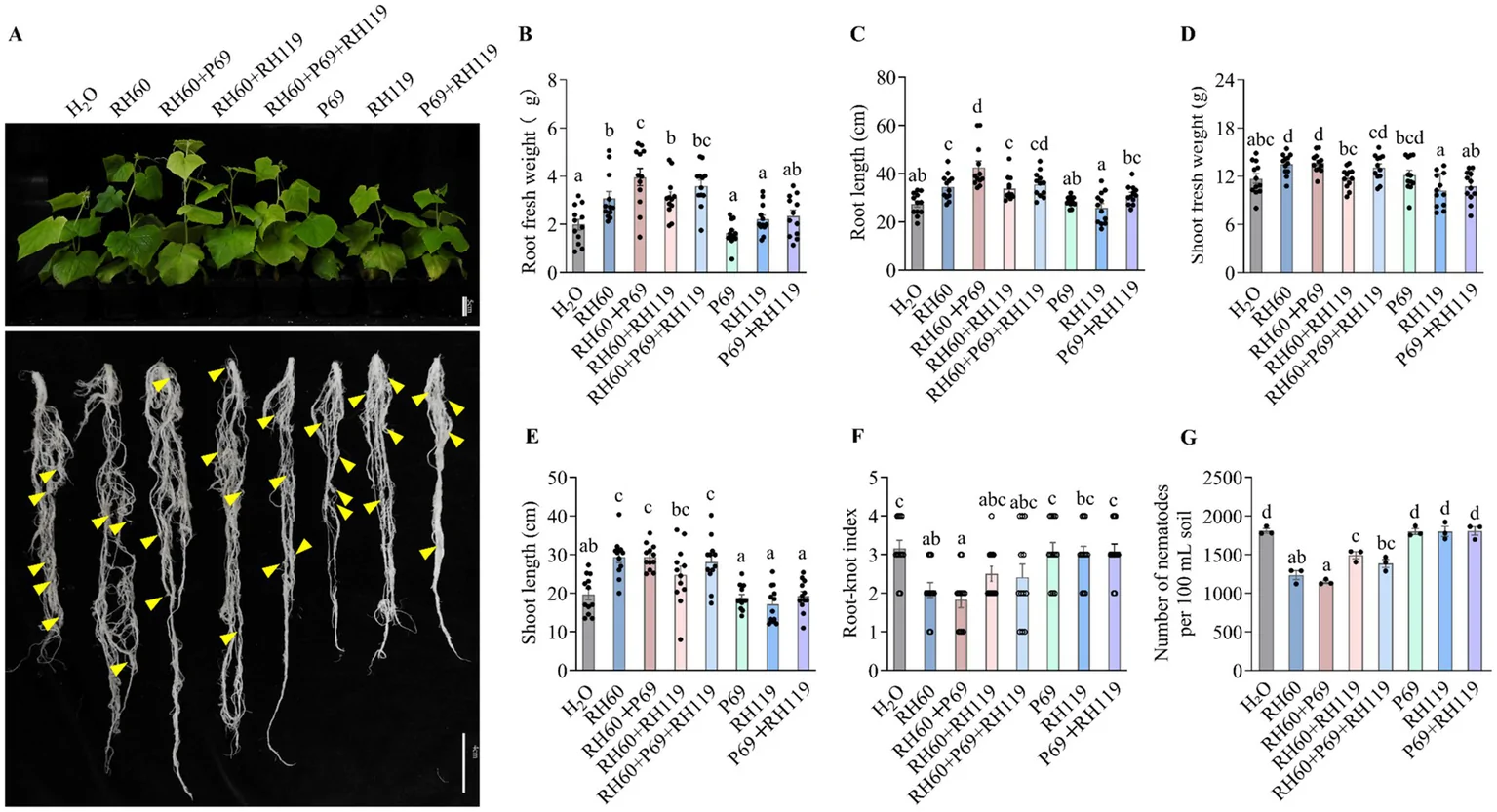
Effects of bacterial treatments on cucumber growth and *M. incognita*-caused disease. Cucumber plants were co-inoculated by different bacterial strains and *M. incognita*, using cucumber plants only inoculated with *M. incognita* as the control (H_2_O). Cucumber plants were sampled **(A)**, and different growth indexes **(B–E)**, root-knot index **(F)**, and number of nematodes in soil **(G)** were recorded at 30 dpi of *M. incognita*. Values are mean ± SE, *n* = 12 **(B–E)** and 3 **(F)**. Different lowercase letters indicate significant differences (one-way ANOVA with post-hoc Tukey HSD test, *p* < 0.05). Bar = 5.0 cm **(A)**.

Subsequently, the impacts of P69 and RH119 on RH60-mediated cucumber growth promotion and nematode disease alleviation were investigated (Figure 5). At 30 dpi of *M. incognita*, co-inoculation of P69 and RH60 significantly increased cucumber root fresh weight and length compared to sole RH60 inoculation (Figures 5B,C). In contrast, RH119 co-inoculation reduced shoot weight (Figure 5D), indicating opposite impacts of P69 and RH119 on RH60-mediated cucumber growth promotion. Additionally, compared to sole RH60 inoculation, P69 co-inoculation showed a decreasing trend in root-knot index and soil nematode population at 30 dpi, although no significant difference was observed at *p* < 0.05 (Figures 5F,G). RH119 co-inoculation exerted the opposite effect to P69, significantly increasing the soil nematode population (Figures 5F,G). Collectively, our results demonstrate that P69 and RH119 play opposite roles in RH60-mediated alleviation of *M. incognita*-caused root-knot nematode disease in cucumber.

### 3.4 P69 and RH119 affect RH60 growth mainly through their VOCs

To determine the metabolites of P69 and RH119 that mediate their impacts on RH60 growth, we first investigated the effects of these metabolites under different treatments on RH60 growth. Results showed that P69 metabolites significantly enhanced RH60 growth, while heat treatment and methanol extraction attenuated this enhancement (Supplementary Figures S5A,C). This indicates that volatile organic compounds (VOCs) in P69 metabolites may play a key role in this process. Similarly, RH119-derived VOCs may contribute to its inhibitory effect on RH60 growth, as heat treatment and methanol extraction reduced this inhibition (Supplementary Figures S5B,D). Two-compartment petri dish co-culture assays further verified these conclusions. The VOCs derived from these two strains exerted the expected effect on RH60 growth compared to the negative control (Supplementary Figure S5E).

**Figure 6:**
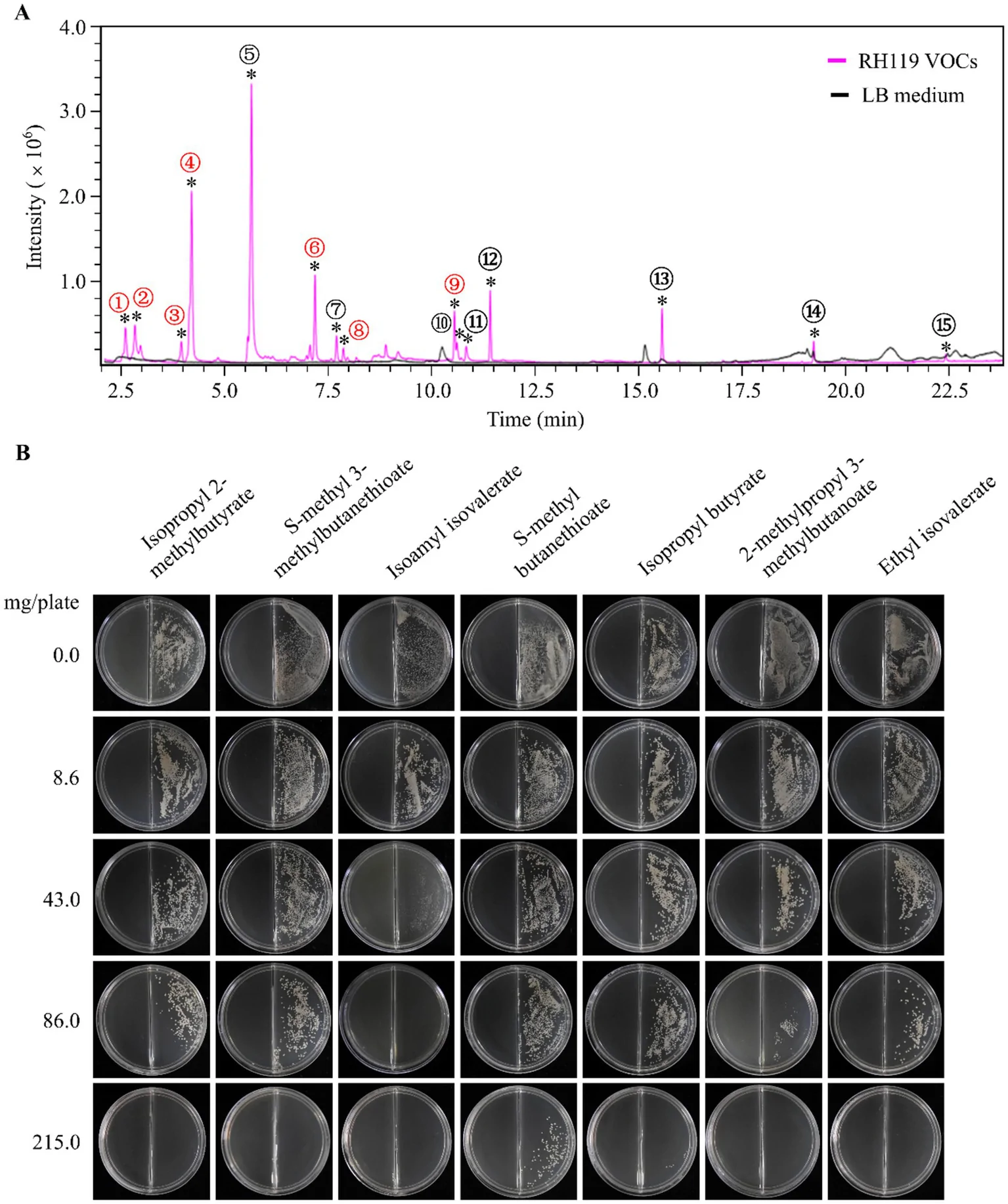
Identification of RH119-derived VOCs and their impacts on RH60 growth. Chromatograms of RH119-derived volatile organic compounds (VOCs) were acquired via GC–MS for identification, with LB medium without RH119 inoculation serving as the negative control **(A)**. Peaks ①–⑮ correspond to the following compounds (in order): Isopropyl butyrate; S-methyl butanethioate; ethyl isovalerate; isopropyl 2-methylbutyrate; 3-methyl-butyric acid isopropyl ester; S-methyl 3-methylbutanethioate; pentanoic acid 1-methylpropyl ester; 2-methylpropyl 3-methylbutanoate; isoamyl isovalerate; valeric acid 4-pentenyl ester; 2-[(trimethylsilyl)oxy]-2-{4-[(trimethylsilyl)oxy]phenyl}ethanamine; dodecamethylcyclohexasiloxane; 3-butoxy-1,1,7,7-hexamethyl-3,5,5-tris(trimethylsiloxy)tetrasiloxane; 3-isopropoxy-1,1,1,7,7,7-hexamethyl-3,5,5-tris(trimethylsiloxy)tetrasiloxane; and 4-fluoro-1-methyl-5-carboxylic acid ethyl ester **(A)**. Among these, the compounds corresponding to the peaks marked in red were further investigated **(A)**. The effects of different RH119-derived VOCs at various concentrations on RH60 growth were evaluated on LA media **(B)**.

Subsequently, RH119-derived VOCs were identified via GC–MS, leading to the detection of 15 components. Based on relative contents, 3-methylbutyric acid isopropyl ester (39.10%), isopropyl 2-methylbutyrate (16.90%), and S-methyl 3-methylbutanethioate (9.31%) ranked as the top three components (Figure 6A; Supplementary Table S3).

The inhibitory effects of seven out of these 15 components on RH60 growth were evaluated using their reference standards. Isoamyl isovalerate exhibited the strongest inhibitory activity, followed by 2-methylpropyl 3-methylbutanoate (Figure 6B). A significant inhibitory effect was observed for all tested components at the assayed concentrations (Figure 6B). Considering the relative contents of these seven components in RH119 VOCs, isoamyl isovalerate (3.84% vs. 8.6 mg/plate) and isopropyl 2-methylbutyrate (16.9% vs. 215.0 mg/plate) contributed the most to VOC-mediated RH60 growth inhibition (Figure 6; Supplementary Table S3). This was followed by S-methyl 3-methylbutanethioate and 2-methylpropyl 3-methylbutanoate (Figure 6; Supplementary Table S3). Ethyl isovalerate (0.86% vs. 86.0 mg/plate) showed the weakest contribution among the seven components (Figure 6; Supplementary Table S3). These results indicate that the VOC-mediated growth inhibition of RH60 is dose-dependent.

## 4 Discussion

### 4.1 General

Continuous cropping obstacles in intensive facility cucumber production have become a critical constraint to sustainable agriculture. Root-knot nematode (*Meloidogyne* spp.) infection is one of the most devastating consequences of rhizosphere ecosystem imbalance (Tian et al., 2020; Zhou et al., 2021). Crop rotation, as an environmentally friendly agronomic practice, has been widely recognized for alleviating such obstacles by reshaping soil microbial communities and optimizing ecological processes (Liu et al., 2023; Kim et al., 2025). However, the underlying microbial interaction mechanisms remain poorly understood. The previous study uncovered that the rhizobacterium *P. oxydans* strain RH60, specifically recruited by pepper root exudates, alleviates *M. incognita*-caused nematode disease in cucumber under a cucumber-pepper rotation system (Tian et al., 2025). However, it remains unknown how cucumbers and peppers restructure the rhizobacterial community and regulate the rhizobacterial interactions through their root exudates to modulate cucumber root-knot nematode disease. This study systematically elucidates the bidirectional regulatory effects of rhizobacterial interactions centered on RH60. These findings provide novel insights into the ecological processes underlying continuous cropping obstacles and rotation-mediated remediation.

### 4.2 Bidirectional regulation of rhizobacterial interaction networks centered on RH60

**Figure 7:**
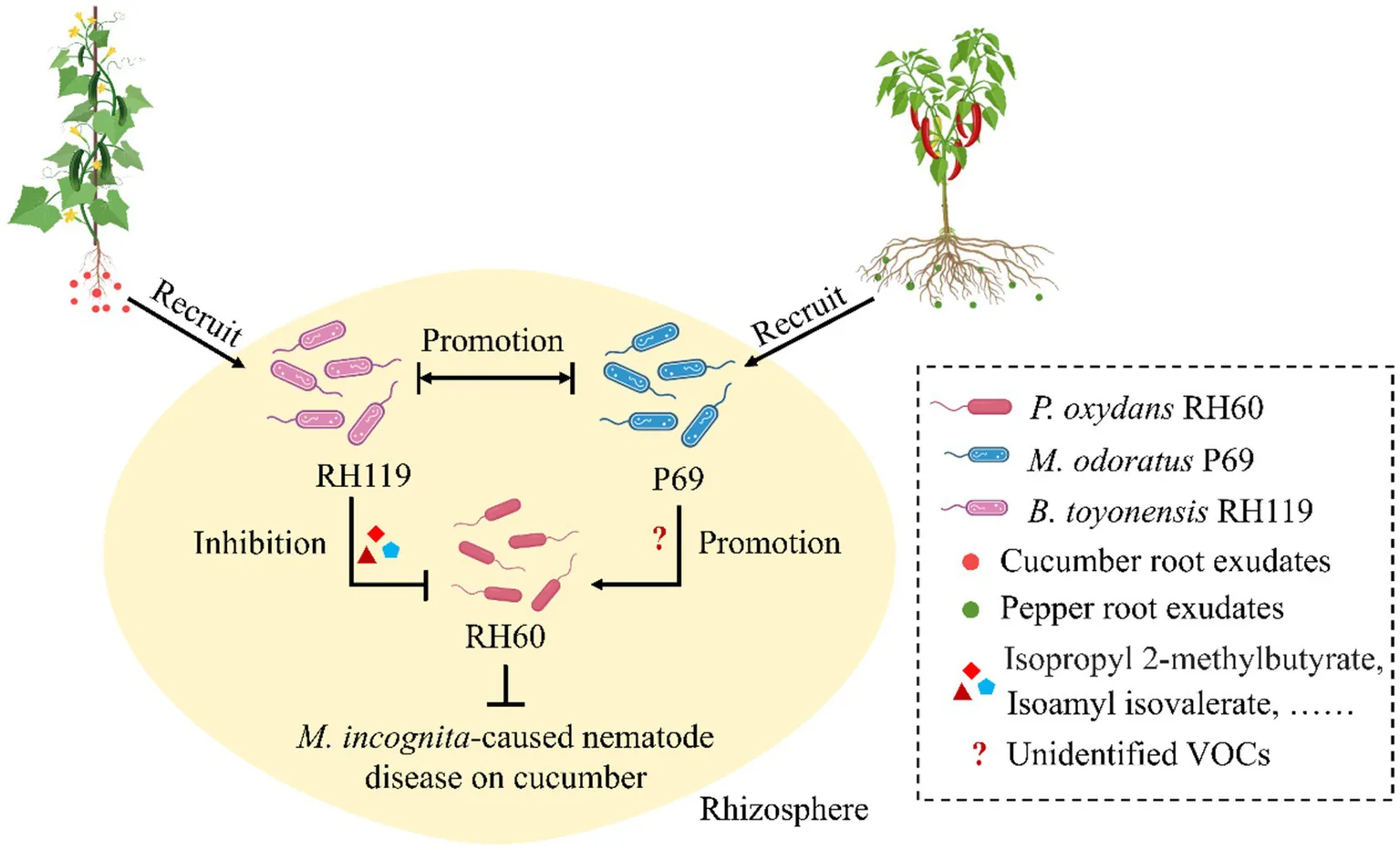
RH60-centered model: bidirectional regulation of cucumber root-knot nematode disease in two different cropping systems. Rhizosphere bacterial strain RH60 plays a pivotal role in alleviating *M. incognita*-caused root-knot nematode disease on cucumber under pepper-cucumber rotation. During pepper cultivation, pepper root exudates specifically recruit rhizobacterium *M. odoratus* strain P69, which enhances RH60’s disease-alleviating effect by promoting RH60 colonization in cucumber rhizosphere. In contrast, under continuous cucumber cropping, cucumber root exudates specifically attract another rhizobacterial strain, RH119 (*B. toyonensis*). RH119 inhibits RH60’s rhizosphere colonization via its VOCs, including isopropyl 2-methylbutyrate and isoamyl isovalerate. This suppression of RH60 colonization impairs its disease-alleviating function, thereby increasing the severity of cucumber root-knot nematode disease and ultimately leading to continuous cropping obstacles in cucumber. Notably, no growth-promoting interactions were detected between strains P69 and RH119.

A pivotal finding of this study is the identification of a RH60-centered rhizobacterial interaction network that mediates opposite disease outcomes under continuous cropping and crop rotation (Figure 7). Pepper rotation recruits the rhizobacterial strain *M. odoratus* P69 through its root exudates, leading to the enrichment of RH60 in cucumber rhizosphere. Consequently, cucumber root-knot nematode disease is alleviated. In contrast, cucumber continuous cropping enriches the rhizobacterial strain *B. toyonensis* RH119, resulting in the inhibition of RH60. Consequently, RH60 function in reducing cucumber nematode disease is impeded. Previous research on rhizobacterial biocontrol has predominantly focused on single-strain effects (Du et al., 2022; Orozco-Mosqueda et al., 2022), while neglecting the complex inter-microbial interactions that shape rhizosphere ecosystem function. Our study reveals that the disease-suppressive capacity of RH60 is not an intrinsic trait. Instead, it is dynamically regulated by its interactions with other rhizobacteria. Under continuous cropping, RH119 is specifically recruited to inhibit RH60, while rotation enriches P69 to synergize with RH60.

Pot experiment data further highlight the superiority of this network-based regulation. Co-inoculation of P69 and RH60 reduced the cucumber root-knot nematode disease and enhanced cucumber root development compared to RH60 alone. The synergized effect of bacterial strains on controlling plant diseases has been widely reported (Saad et al., 2020; Zhou et al., 2022; Qiao et al., 2024). Conversely, RH119 co-inoculation impeded RH60-mediated alleviation of this nematode disease. This finding is consistent with the reports that continuous cropping enriches antagonistic microbes that compromise beneficial bacterial function (Xiong et al., 2015; Feng et al., 2022). This bidirectional regulatory mechanism strongly validates our initial hypotheses and fills a critical gap in understanding the role of crop rotation in reshaping microbial interaction networks.

Notably, under our pot conditions, several phenotypic indices showed no statistically significant differences between RH60 single inoculation and RH60-P69 co-inoculation. These indices include shoot length, root-knot index, and nematode density in soil. RH60 exerts nematode-suppressive capacity in an abundance-dependent manner (Tian et al., 2025). Although P69 significantly elevates RH60 rhizosphere colonization, the increased RH60 population may not reach the functional threshold required to trigger statistically detectable changes in these specific disease and shoot-growth metrics. By contrast, significant alterations were observed for root fresh weight and length, which exhibit higher sensitivity to RH60 population fluctuation. Such discrepancy among different measurement indices highlights that P69 and RH119 function as upstream modulators of RH60 rhizosphere fitness rather than direct nematode-suppressive agents.

### 4.3 Innovative “dual-linkage” mediated by root exudates and VOCs

A key innovation of this study is the discovery of a “dual-linkage” mechanism, wherein plant-derived root exudates and microbe-derived VOCs synergistically mediate RH60-centered rhizobacterial interactions. This kind of integration rarely reported in prior researches. Most studies have independently investigated root exudates or microbe-derived VOCs as discrete signaling pathways. For example, it was reported that root exudates from continuously cropped peanuts reduce disease-suppressive rhizosphere bacteria (Zhou Y. Y. et al., 2023). This reduction impairs soil’s inhibition of *F. oxysporum* and exacerbating root rot, whereas peanut root exudates from crop rotation promote the enrichment of such bacteria (Zhou et al., 2023b). But this work does not link this recruitment to subsequent microbial metabolite interactions. In contrast, our study demonstrates that root exudates act as key regulators mediating bacterial interactions through precise selective recruitment. Cucumber root exudates specifically enriched strain RH119, thereby facilitating the accumulation of RH119-derived RH60-inhibitory VOCs in continuous cropping soil (Figure 7). Conversely, pepper root exudates preferentially recruit strain P69, which in turn enhances RH60’s rhizosphere colonization and disease-suppressive capacity through its VOCs (Figure 7). This selective recruitment reflects a plant adaptive strategy to environmental stress. Continuous cropping-induced autotoxicity and nematode pressure drive cucumbers to inadvertently enrich antagonistic bacteria (RH119). In contrast, rotation with pepper redirects the rhizobacterial community toward beneficial synergists (P69). This finding extends the current theory of plant-microbe crosstalk. We demonstrate that plants can modulate microbial interaction networks through root exudates. This mechanism goes beyond merely recruiting individual beneficial microbes, as described previously (Santoyo, 2022; Liu et al., 2024; Vanin et al., 2025). Furthermore, our identification of key ester-type VOCs from RH119 expands the known ecological functions of microbe-derived volatiles. Among the 15 detected VOCs, isoamyl isovalerate (3.84% relative content) and isopropyl 2-methylbutyrate (16.90% relative content) exhibited the strongest inhibitory effects on RH60 in a concentration-dependent manner. Prior studies have linked these VOCs to plant-fungal interactions and insect oviposition attraction (Guo et al., 2023; Mobarak et al., 2025), but this is the first report of their role in mediating rhizobacterial antagonism. Notably, 3-methylbutyric acid isopropyl ester accounted for 39.10% of RH119’s VOC profile (the highest relative content). However, its biological function remains uncharacterized due to the lack of a reference standard. This compound represents a promising direction for future research.

### 4.4 Practical implications for sustainable agriculture

The synergistic interaction between P69 and RH60 offers dual benefits for agricultural production by reducing nematode disease severity and enhancing cucumber root growth. This addresses both disease control and yield improvement. Previous studies have reported that single-strain biocontrol agents often exhibit unstable efficacy under field conditions. This instability is due to poor rhizosphere colonization and vulnerability to indigenous microbial competition (Zhang et al., 2023). Synergistic effects exist among multiple strains in the control of plant nematode diseases (Albaghdady et al., 2025; Rashidifard et al., 2025). The rotation-mediated RH60-P69 synergistic system lays the foundation for developing composite biofertilizers, which could directly mitigate nematode damage in continuous cropping systems. From a breeding perspective, our findings align with the proposal that modifying root exudate profiles to optimize microbial recruitment is a viable strategy for disease resistance (Kawasaki et al., 2021; Zhang and Mason, 2022; Stirnemann and Sasse, 2025). Specifically, modifying cucumber root exudates could reduce RH119 recruitment or enhance P69 attraction. This approach could integrate genetic improvement with ecological management, offering a proactive solution to continuous cropping obstacles.

### 4.5 Limitations and future directions

While pot experiments validate the core mechanism, field validation is essential to confirm its applicability under complex natural conditions. Soil type, climate factors, and indigenous microbial communities may modulate the interactions between RH60, P69, and RH119, potentially affecting the efficacy of nematode suppression. Future field trials should evaluate the performance of the P69-RH60 composite inoculant across different soil types and cropping systems. Additionally, the root-exudate constituents responsible for recruiting P69 or RH119 and the P69-originated VOCs enhancing RH60 growth are yet to be identified. Uncovering these compounds may support the design of synthetic root-exudate and VOC mimics. These mimics could artificially reshape the rhizobacterial community, as demonstrated by Pantigoso et al. (2023) and Luo et al. (2025) for recruiting phosphate-solubilizing bacteria.

## 5 Conclusion

This study clarifies that RH60-centered rhizobacterial networks bidirectionally regulate cucumber root-knot nematode disease. Continuous cropping recruits RH119 via cucumber root exudates, and RH119-derived VOCs (isoamyl isovalerate, etc.) inhibit RH60 to impede RH60 function. Cucumber-pepper rotation recruits P69 via pepper root exudates, and P69 synergizes with RH60 to enhance disease suppression and root growth. A key highlight is the “dual-linkage” mechanism. In this mechanism, root exudates recruit specific bacteria and microbe-derived VOCs mediate their interaction with RH60. This study advances the understanding of rotation-microbe interplay, and provides preliminary theoretical evidence for developing composite biofertilizers and optimizing root exudate regulation.

## Data Availability

The authors confirm that the data supporting the findings of this study are available within the article and its Supplementary material.

## Supplementary material

SUPPLEMENTARY FIGURE S1 The technical roadmap of this study.

SUPPLEMENTARY FIGURE S2 Assessing the recruitment of seven RH60 inhibitory and three RH60 growth-promoting strains by cucumber and pepper root exudates. Root exudates (100 μg/mL) from cucumber (for RH60 inhibitory strains) and pepper (for RH60 growth-promoting strains) were used to assess three indices: (1) chemotactic responses (2 h incubation, CFU counting); (2) strain growth (12 h culture, OD_600_ detection); (3) biofilm formation (24 h incubation, OD_562_ detection). Values are mean ± SE (*n* = 6 for growth promotion, *n* = 5 for biofilm formation). *, ** and *** indicate significant differences (Student’s *t*-test) at *p* < 0.05, *p* < 0.01 and *p* < 0.001, respectively. “ns” indicates no significant difference (Student’s *t*-test) at *p* < 0.05.

SUPPLEMENTARY FIGURE S3 Identification of RH60 growth-promoting strain P69 and inhibitory strain RH119. Phylogenetic trees of P69 (**A**, growth-promoting) and RH119 (**B**, inhibitory) were constructed based on 16S rRNA gene sequences via the neighbor-joining method with 1,000 bootstrap replicates, using reference sequences from NCBI (accession numbers in parentheses). Gram staining and colony morphologies of the two strains are also shown **(A,B)**. Bar = 5 μm.

SUPPLEMENTARY FIGURE S4 Standard curve between RH60 copy number and Ct values. A TA-cloning vector harboring the RH60 genomic DNA fragment (amplified with RH60 species-specific PCR primers) was used as the DNA template. qPCR was performed with serial concentrations of this vector (representing different RH60 copy numbers) and the aforementioned species-specific primers to obtain corresponding Ct values. The standard curve was constructed by linear regression of RH60 copy numbers against Ct values (Y = −3.3232X + 39.649; R^2^ = 0.9964). Data are presented as mean ± SEM, *n* = 3.

SUPPLEMENTARY FIGURE S5 Physicochemical characterization of active components from strains P69 and RH119 affecting RH60 growth. Effects of P69 **(A)** and RH119 **(B)** on RH60 growth were evaluated on LA media. OD_600_ values of RH60 cultures in LB medium supplemented with three differently treated fermentation filtrates of P69 **(C)** or RH119 **(D)** were recorded after 16 h incubation at 28 °C, using LB medium without fermentation filtrates as the control. In addition, two-compartment Petri dish co-cultivation assay were performed to determine the impact of VOCs from P69 and RH119 on RH60 growth **(E)**, utilizing LB medium replacing P69/RH119 suspensions as the control. Values are mean ± SE, *n* = 6, and different lowercase letters indicate significant differences (two-way ANOVA followed by Tukey HSD post-hoc test) at *p* < 0.05 **(C,D)**.
